# Nurses’ motivation for performing cardiopulmonary resuscitation: a cross-sectional study

**DOI:** 10.1186/s12912-024-01853-9

**Published:** 2024-03-15

**Authors:** Mozhdeh Najafi, Safoura Yadollahi, Mahboobeh Maghami, Ismail Azizi-Fini

**Affiliations:** 1 Trauma Nursing Research Center, Department of Critical Care Nursing and Emergency, Kashan University of Medical Sciences, Kashan, Iran; 2 Department of Biostatistics and Epidemiology, Isfahan University of Medical Sciences, Isfahan, Iran

**Keywords:** Motivation, Nurse, Cardiopulmonary resuscitation, Emergency, Attitude

## Abstract

**Background:**

Nurse motivation can have a significant impact on the quality of cardiopulmonary resuscitation and the patients’ survival. Therefore, the present study aimed to examine nurses’ motivation for performing cardiopulmonary resuscitation.

**Methods:**

This cross-sectional study focused on 217 nurses in a teaching hospital in Iran, in 2023. A random sample of nurses was selected from four hospital departments (emergency, critical care, medical, and surgery). These nurses completed the demographic information and motivation for cardiopulmonary resuscitation questionnaires. The data were analyzed using Mann-Whitney, Spearman coefficients, and Kruskal-Wallis and multiple linear regression tests.

**Results:**

The mean score of the dimension of the feeling of achievement (4.10 ± 0.50) was high in the nurses’ motivation for performing cardiopulmonary resuscitation. There were more motivational factors in the emergency department compared to the other departments in terms of the feeling of achievement, high chance of success, low chances of success, recognition and appreciation, perceived importance, and beliefs (*p* < 0.05). The nurses who had participated in cardiopulmonary resuscitation workshops and had a bachelor’s degree had a higher mean score in the dimension of perceived importance (*p* < 0.05). The correlation coefficient showed that there was a significant negative correlation between the nurses’ frequency of participation in cardiopulmonary resuscitation and their motivation scores in the dimensions of the feeling of achievement(*r*=-0.170), low chances of success(*r*=-0.183), perceived importance (*r* = -0.302), and beliefs (*r* = -0.250; *p* < 0.05). The department variable predicted the motivation score in the dimensions of feeling of achievement, high chance of success, low chance of success, perceived importance, and beliefs. The sex variable predicted the motivation score in the dimensions of facilitator of resuscitation and high chance of success. Besides, the variable of years of membership in the CPR team predicted the motivation score in the feeling of achievement and high chance of success (*p* < 0.05).

**Conclusion:**

Nurses would be more motivated to perform a quality cardiopulmonary resuscitation if they had a feeling of success. The nurses’ motivation was affected by certain factors such as their department, sex, education level, years of membership in CPR team, number of participation in CPR, and participation in educational workshops.

## Key Points

• Despite the improvement in CPR guidelines, survival rates remain low.

• A feeling of success in CPR increases nurses’ motivation.

• A high level of motivation leads to high-quality CPR.

## Introduction

Work motivation is an important measure of healthcare professionals’ response to the increasing challenges and demands in healthcare organizations [1]. Motivation is one of the most important issues related to organizational behavior. Therefore, managers should be aware of their employees’ motivation to determine the reasons behind their' behavior [2]. The human resources constitute the most important capital of the organization. Therefore, it is necessary for the managers to obtain information about their motivation [3]. This issue stems from the existence of differences among individuals in terms of their job-related abilities and job-performance reasons (i.e. motivation). Motivation is a state that persuades people to exhibit a certain behavior. That is, it is the basic factor that shapes people’s activities and efforts [4]. In fact, motivation has certain effects on the individuals’ direction of behavior, along with intensity, effort, and continuity of behavior [5]. Maslow’s hierarchy of needs theory [6], Herzberg’s two-factor theory [2, 7], and the theory of Existence, Relatedness and Growth [8]are among the content theories related to motivation. These theories focus on the factors that affect motivation. Moreover, equity theories [9], attribution theory [10], expectancy theory [11], and goal setting theory [12] emphasize the flow of motivation. Some factors including the number and depth of massage, return of the chest to its original state, prevention of excessive ventilation are the factors that influence the quality of cardiopulmonary resuscitation, and are are affected by the resuscitators’ performance [13]. Nonetheless, the performance of health workers, including nurses, is affected by their motivation. Furthermore, motivation affects the continuity of their performance. This issue has been associated with the increase in the spontaneous return of blood to the patients’ brain and the return of patients to life in cardiopulmonary resuscitation [4]. In general, nursing staff are the first group of health workers that deal with the cardiac arrest patients. Therefore, their work motivation is a key factor in their success in increasing the spontaneous return of blood to these patients’ brain and the return of the patients to life [14]. However, there is not adequate information on nurses’ work motivation as a response to increased healthcare demands such as CPR [1, 15]. Healthcare professionals’ motivation and attitude may influence their behavior in CPR situations [16].

## Background

Motivational readiness is associated with significantly lower symptoms of emotional distress in cardiac emergencies and with fewer psychosocial barriers [17]. Consequently, highly motivated rescuers are likely to perform high-quality CPR [18].

Every year, more than 500,000 people die from cardiac arrest [19]. Cardiac arrest must be treated immediately since any delay decreases the patients’ survival chances [16]. The success of CPR is one of the most important indicators of hospital performance [20]. Therefore, the increase in this number indicates the satisfactory performance of the CPR team. CPR has been used as a cardiac arrest treatment for more than five decades. Nonetheless, there have not been any noticeable improvements in the patients’ survival rates after cardiac arrest [21]. Despite constant scientific refinement of guidelines for CPR, only 6–9% of patients survive after their cardiac arrest [22]. The adults’ overall mortality rate after in-hospital cardiac arrest is reported to be 78% [23]. A recent meta-analysis showed that the pooled survival rate at discharge was 15.0% [24]. Moreover, another study showed that one-year survival after in-hospital cardiac arrest was poor (10.7%) [25]. The results of a study in Iran showed that 36.7% of CPRs were successful [20]. The success of CPR is influenced by a variety of rescue-related factors, including technical, communication, management skills, previous experiences, fatigue, and motivation for CPR [26].

According to studies, motivation contributes not only to competence and endurance in CPR but also to appropriate interventions and effective CPR [27]. High-quality CPR improves patient survival [28]. Furthermore, work motivation leads to high-quality performance and better clinical outcomes [29]. The assumption that motivation affects rescuers’ performance and CPR outcomes suggests that the selection of highly-motivated rescuers for CPR is necessary [27, 30]. There is not adequate information on nurses’ work motivation [31]. The results of a systematic review showed that female students’ motivation for attend CPR training courses was high [22]. Another study showed that in the general population, there were four major motivational barriers to the performance of CPR and including fear of legal consequences, emotional issues, knowledge, and situational concerns [32]. According to Kleinman et al., clinical experts do not resuscitate the patients who do not return to life spontaneously in the early stages of CPR due to their poor prognosis of the patients’ return to life [33]. According to Assaroudi et al. [18], resuscitators’ motivations for performing CPR are influenced by a number of factors such as the probability of resuscitation success, the resuscitator’s self-efficacy, the possibility of expected results, the possibility of performing CPR interventions, and the possibility of errors. According to them, beliefs and consequences are valuable. In a study that was conducted by Fallahi et al., nursing professionals stated that cardiopulmonary resuscitation was useless for some patients. Moreover, they noted that and the resuscitation of patients with advanced diseases only resulted in their suffering [34]. According to another study, student nurses would start resuscitation outside the hospital if they lacked experience and knowledge [35]. Several studies have investigated the attitudes and motivation of the general public and the students towards learning and performing resuscitation outside the hospital [35–38]. Notwithstanding, few studies have examined the nurses’ motivation. Considering the importance of patients’ survival after CPR and the lack of adequate literature on this issue, the researchers conducted a study to examine the nurses’ motivation for performing CPR and the factors that affected the nurses’ motivation.

## Methods

### Study design

This cross-sectional study focused on the nurses in X, Iran.

### Sample

Systematic random sampling was used to select 217 nurses from among the nurses who worked in an educational hospital from January to April 2023. Based on the variance of the motivation score in a previous study ($\delta$ =1.03) [31], and α = 0.05, d = 0.135, the sample size was estimated to be 160. However, considering the possible 6% dropout of participants, we selected 217 nurses.

$$ n=\frac{{\left({z}_{1-\raisebox{1ex}{$a$}\!\left/ \!\raisebox{-1ex}{$2$}\right.\delta }\right)}^{2}}{{d}^{2}}\cong 217$$

The inclusion criteria involved: having at least three months of work experience and having participated in CPR at least once. On the other hand, the participants who did not complete the questionnaires were excluded from the study.

### Data collection

Data were collected using a demographic questionnaire and a cardiopulmonary resuscitation motivation scale (CPRMS). After obtaining the consent to the study from the hospital authorities, first, the researchers received a list of nurses from the nursing office, and selected the nurses. Second, they administered the questionnaire to them in a private setting. The nurses completed the questionnaire on their own.

### Instrument

A demographic questionnaire was administered to the participants. This instrument asked the participants to provide information on their age, sex, work history, education level, hospital department, number of children, number of shifts per month, number of CPRs (approximate total working time), duration of membership in the CPR team (year), and experience of participating in CPR workshops.

A second component of the instrument was the CPRMS, which was developed and psychometrically evaluated by Assaroudi et al. in Iran. There were 43 items in the CPRMS, and it is a valid and reliable instrument for measuring CPR motivation. This instrument examined eight dimensions including: facilitators of resuscitation (6 items), feeling of achievement (7 items), high chances of success (8 items), low chances of success (6 items), recognition and appreciation (6 items), accountability (4 items), perceived importance (3 items), and beliefs (3 items). The scale of the questionnaire was a five-point Likert scale. More specifically, the first three items were rated on a scale ranging from *always* to *never* (i.e. 5 = always, 4 = most of the time,3 = sometimes,2 = rarely, and 1 = never). Moreover, the remaining items were rated on a scale ranging from *completely agree* to *‘neither agree nor disagree* (i.e. 5 = I completely agree score, 4 = I agree, 3 = I disagree, 2 = I completely disagree, and 1 = neither agree nor disagree). The content validity index of the instrument was 0.97. Furthermore, Cronbach’s alpha measure showed that its internal consistency was 0.92 [18]. To assess the instrument’s reliability, ten nurses who are members of the CPR team completed it. They then completed the tool again after a week. A Cronbach’s alpha of 0.94 was calculated.

The questionnaire did not have a total score and the score of each dimension was calculated separately. The minimum and maximum scores in the dimension of facilitators of resuscitation were 6 and 30 respectively. Moreover, these scores ranged from 7 to 35 in the case of feeling of achievement dimension. Furthermore, the minimum and maximum scores of high chances of success dimension were in the range of 8 to 40. In addition, these scores ranged from 6 to 30 in regard to low chances of success dimension. Additionally, the relevant scores were in the range of 6 to 30 in the case of recognition and appreciation dimension. Besides, the relevant score ranged from 4 to 20 for the accountability dimension and from 3 to 15 for the perceived importance dimension. Lastly, the minimum and maximum scores of the beliefs dimension were in the range of 3 to 15 c. Higher scores in each dimension indicated higher motivation of the reviver in the relevant dimension.

### Data analysis

SPSS Version 16.0 (SPSS Inc., Chicago, IL, USA) was used to analyze the data. The results were provided as the mean ± standard deviation or frequency (percentage). Nonparametric Mann‒Whitney and Kruskal‒Wallis tests were used to perform the data analysis since the assumption of normality was violated. Spearman’s correlation tests were used to examine the relationships between the relevant variables. A multiple linear regression model was fitted separately for each dimension of the questionnaire. Statistical significance was considered to be *p* < 0.05.

### Ethical considerations

Informed consent was obtained from all of the participants. Moreover, the participants were assured of the confidentiality of their personal information. All of the participants’ rights were protected based on to the latest version of the Helsinki Declaration.

## Results

A total of 214 nurses were selected from among the nurses in an educational hospital. Nonetheless, three nurses were excluded due to the fact that they did not complete the questionnaires. In this study, the mean participant age was 30.70 ± 5.08 years. Moreover, the mean experience of the CPR team was 148.71 ± 125.32. Furthermore, the number of shifts per month was 26.87 ± 2.64. Lastly, the mean work experience was 6.48 ± 5.08 years (Table 1).

**Table 1:** Nurses’ characteristics

variable	Mean ± SD	Maximum	Minimum
Age (year)	30.70 ± 5.08	50	22
Work experience (year)	6.48 ± 5.08	26	1
Shift a month (number)	26.87 ± 2.64	40	17
Children (number)	0.47 ± 0.76	4	0
Experience of CPR (number)	148.71 ± 125.32	600	1
Membership in the CPR team (year)	6.33 ± 5.078	26	1

Approximately 51.9% (*n* = 111) of these nurses were female, and 92.1% (*n* = 197) of them had attended CPR workshops in the past. The numbers of items varied from dimension to dimension. Therefore, first, the mean score for each dimension was divided by the number of items in that dimension. Second, the result was compared with the results of the other dimensions. The results indicated that the highest mean score was the mean of the feeling of achievement in CPR (4.10 ± 0.50) dimension. On the other hand, the lowest mean score was with the mean of the dimension of low chances of success in CPR (3.20 ± 0.72). An examination of the other dimensions revealed the following mean scores: facilitators of resuscitation: 3.77 ± 0.60; high chances of success: 4.02 ± 0.56; recognition and appreciation: 3.64 ± 0.64; accountability: 3.79 ± 0.63; perceived importance: 4.08 ± 0.67; and beliefs 3.98 ± 0.73. The result of the Kruskal‒Wallis test showed that there was a statistically significant difference between the different departments in terms of the nurses’ feeling of achievement (*p* < 0.0001), high chances of success (*p* = 0.002), low chances of success (*p* < 0.001), perceived importance (*p* < 0.0001), and beliefs (*p* = 0.005). In this way, nurses working in the emergency department scored higher in all of the above dimensions than nurses who work in the medical, critical care, and surgery departments. Moreover, the result of Mann‒Whitney test indicated that there were significant differences between the mean scores of perceived importance (*p* = 0.047) and beliefs (*p* = 0.047) based on previous participation in a CPR workshop. Consequently, nurses who have participated in CPR workshops in the past scored higher in the dimension of perceived importance and beliefs than nurses who have not participated. According to the results of this test, there was a significant statistical difference between nurses with a bachelor’s degree and nurses with a master’s degree in the dimension of perceived importance (*p* = 0.003; Table 2).

**Table 2:** Mean CPR motivation score according to the nurses’ characteristics

Variable	Total score	Sex		Level of Education		Department				CPR Workshop	
		Male	Female	Bachelor’s degree	Master’s degree	Emergency	Critical care	Surgery	Medical	Yes	No
n(%)	214(100)	103(48.1)	111(51.9)	152(71)	62(29)	54(25.2)	74(34.6)	46(21.5)	40(18.7)	197(92.1)	17(7.9)
Dimension	Mean ± SD	Mean ± SD	Mean ± SD	Mean ± SD	Mean ± SD	Mean ± SD	Mean ± SD	Mean ± SD	Mean ± SD	Mean ± SD	Mean ± SD
**Facilitators of resuscitation**	3.77 ± 0.60	22.0 ± 4.03	23.1 ± 3.17	22.3 ± 3.91	23.3 ± 2.77	21.9 ± 4.8	23.06 ± 2.9	22.26 ± 3.6	23.35 ± 2.7	22.66 ± 3.6	22.59 ± 4.0
*P* value	-	0.057		0.21		0.21				0.91	
**Feeling of achievement**	4.10 ± 0.50	28.5 ± 3.13	28.8 ± 3.81	28.7 ± 3.74	28.5 ± 2.82	30.7 ± 3.54	28.1 ± 3.17	28.1 ± 2.82	27.6 ± 3.77	28.6 ± 3.41	29.5 ± 4.38
*P* value	-	0.43		0.35		0.0001				0.56	
**High chances of success**	4.02 ± 0.56	32.8 ± 3.78	31.6 ± 5.09	32.3 ± 4.69	32.0 ± 4.16	33.6 ± 5.82	32.3 ± 4.35	31.4 ± 3.49	31.1 ± 3.44	32.2 ± 4.49	32.2 ± 5.1
*P* value	-	0.08		0.57		0.002				0.88	
**Low chances of success**	3.20 ± 0.72	19.1 ± 4.13	19.2 ± 4.51	19.4 ± 4.46	18.5 ± 3.90	21.3 ± 5.53	18.5 ± 3.51	19.0 ± 3.65	17.8 ± 3.51	19.1 ± 4.32	20.0 ± 4.39
*P* value	-	0.95		0.11		0.001				0.41	
**Recognition and appreciation**	3.64 ± 0.64	21.8 ± 3.86	21.9 ± 3.88	21.9 ± 4.08	21.8 ± 3.27	21.9 ± 4.98	21.7 ± 3.74	22.4 ± 3.39	21.9 ± 2.84	21.9 ± 3.83	21.7 ± 4.3
*P* value	-	0.88		0.75		0.88				0.98	
**Accountability**	3.79 ± 0.63	15.2 ± 2.33	15.0 ± 2.70	15.1 ± 2.71	15.1 ± 2.01	15.5 ± 3.50	14.8 ± 1.94	15.1 ± 2.62	15.2 ± 1.66	15.0 ± 2.38	16.0 ± 3.82
*P* value	-	0.71		0.72		0.24				0.06	
**Perceived importance**	4.08 ± 0.67	12.1 ± 1.97	12.36 ± 2.07	12.4 ± 2.01	11.6 ± 1.95	13.4 ± 1.88	11.5 ± 2.17	12.2 ± 1.61	11.9 ± 1.60	13.1 ± 2.01	12.1 ± 2.02
*P* value	-	0.25		0.003		0.0001				0.047	
**Beliefs**	3.98 ± 0.73	11.68 ± 2.30	12.18 ± 2.06	11.9 ± 2.33	11.9 ± 1.80	12.7 ± 2.31	11.8 ± 2.33	11.5 ± 2.03	11.5 ± 1.69	12.7 ± 2.15	11.3 ± 2.51
*P* value	-	0.10		0.54		0.005				0.047	

There were significant negative correlations between the mean scores of motivation in the dimensions of the feeling of achievement (*r* = -0.170), low chance of success (*r* = -0.183), perceived importance (*r* = -0.302), and beliefs (*r* = -0.250) and the number of CPRs performed during the nurses’ working career (*p* < 0.05; Table 3).

**Table 3:** Correlation between the mean CPR motivation score and nurses’ characteristics

Variables Dimension	Membership in the CPR team (year)	CPR (number)	Children (number)	shifts per month (number)	Work experience (year)	Age(year)
	r; p	r; p	r; p	r; p	r; p	r; p
Facilitators of resuscitation	0.12; 0.07	0.10; 0.12	0.19; 0.25	0.02; 0.75	0.17; 0.21	0.12; 0.06
Feeling of achievement	0.04; 0.48	-0.17; 0.01	0.002; 0.98	-0.08; 0.23	0.01; 0.78	-0.03; 0.59
High chances of success	0.09; 0.18	-0.07; 0.29	-0.03; 0.60	0.01; 0.87	0.05; 0.47	0.03; 0.65
Low chances of success	0.01; 0.84	-0.18; 0.007	0.01; 0.84	-0.02; 0.70	0.003; 0.96	-0.01; 0.80
Recognition and appreciation	0.02; 0.73	-0.04; 0.51	0.10; 0.13	0.04; 0.55	0.04; 0.53	-0.002; 0.97
Accountability	-0.04; 0.51	-0.12; 0.06	0.01; 0.86	-0.02; 0.71	-0.05; 0.41	-0.08; 0.21
Perceived importance	-0.04; 0.49	-0.30; 0.0001	-0.16; 0.11	0.06; 0.35	-0.08; 0.21	-0.12; 0.07
Beliefs	-0.01; 0.78	-0.25; 0.0001	0.001; 0.99	0.03; 0.58	-0.02; 0.75	-0.03; 0.63

All coefficients are based on the Spearman correlation coefficient.

An analysis of backward multiple linear regression was conducted in this study. The model included all study variables in each dimension as predictors. As a result, we eliminated the predictors that had a *p*-value greater than 0.05 step by step. Ultimately, the model that performed best both statistically and clinically was selected for each dimension. Regarding the type of department, the emergency department was considered as a reference and the scores were compared with the emergency department. In the facilitator of resuscitation dimension, only sex has become significant, and a lower facilitator score is found among males (B= -1.09; *p* = 0.023). In the dimension of feeling of achievement, the type of department, age, and years of CPR team membership were significant (*p* < 0.05). In addition, the surgical, medical, and critical care departments have lower achievement scores than the emergency departments. Furthermore, as one’s age increases, their feeling of achievement score decreases. Membership in the CPR team has a positive effect on their achievement score (B = 0.18; *p* = 0.01). As the dimension of the low chance of success has become increasingly important, working in the surgical, medical, and critical care departments has been associated with a lower score (*p* < 0.05). It is important to note that, in the dimension of perceived importance, the type of department and the number of shifts per month have an impact, so that working in surgery and medical departments has a lower score and more shifts have a positive impact (B = 0.13; *p* = 0.005).

There is a significant relationship between the type of department in the belief dimension, so employees in the surgical and medical departments have a lower score in this dimension. The belief score is negatively influenced by the number of CPR (B= -0.003, *p* = 0.04; Table 4).

**Table 4:** Multiple linear regression model for dimensions of CPR motivation questionnaire

Dimension	Predictors	Unstandardized B	Coefficient Std. Error	CI (lower)	CI (upper)	*p*-value
Facilitators of resuscitation	Sex (male)	-1.09	0.48	-2.04	-0.14	0.023
Feeling of achievement	Department(surgery)	-2.62	0.65	-3.89	-1.34	< 0.0001
	Department(medical)	-3.05	0.67	-4.38	-1.72	< 0.0001
	Department (critical care)	-2.42	0.58	-3.57	-1.26	< 0.0001
	Department (emergency)^*^	-	-	-	-	-
	Age	-0.15	0.07	-0.30	-0.006	0.041
	Membership in the CPR team (year)	0.18	0.07	0.04	0.33	0.012
High chances of success	Department(surgery)	-2.15	0.85	-3.82	-0.47	0.01
	Department(medical)	-2.53	0.89	-4.28	-0.79	0.004
	Department (critical care)	-1.19	0.76	-2.69	0.31	0.12
	Department (emergency)^*^	-	-	-	-	-
	Membership in the CPR team (year)	0.24	0.06	0.11	0.37	< 0.0001
	Sex (male)	-1.39	0.58	-2.53	-0.24	0.01
	Children (number)	-1.29	0.45	-2.19	-0.40	0.004
Low chances of success	Department(surgery)	-2.32	0.82	-3.93	-0.70	0.005
	Department(medical)	-3.58	0.85	-5.26	-1.91	< 0.0001
	Department (critical care)	-2.86	0.73	-4.30	-1.42	< 0.0001
	Department (emergency)^*^	-	-	-	-	-
Perceived importance	Department(surgery)	-1.42	0.37	-2.15	-0.68	< 0.0001
	Department(medical)	-1.58	0.38	-2.33	-0.83	< 0.0001
	Department (critical care)	-2.20	0.33	-2.87	-1.54	< 0.0001
	Department (emergency)^*^	-	-	-	-	-
	shifts per month (number)	0.13	0.04	0.04	0.23	0.005
Beliefs	Department(surgery)	-1.09	0.42	-1.93	-0.25	0.01
	Department(medical)	-0.91	0.45	-1.80	-0.02	0.04
	Department (critical care)	-0.54	0.40	-1.34	0.25	0.18
	Department (emergency)^*^	-	-	-	-	-
	CPR(number)	− 0.003	0.0013	− 0.005	-0.0005	0.045

* Emergency department as a reference

## Discussion

In the present study, participants had the highest scores on the feeling of achievement dimension. In other words, having a sense of success during task performance and achieving personal goals were beneficial for nurses and increased their motivation for performing cardiopulmonary resuscitation. A person is forced to exert maximum effort to accomplish his/her desired goals due to his/her need for success [39]. Likewise, another study reported that achievement was a motivating factor in the workplace [7]. According to the findings of another study, nursing staff considered achievement to be the most important motivational factor [40, 41]. According to the interpretation of the present findings, it can be concluded that nurses’ motivation in this dimension was high due to their feeling of being valuable and useful in saving a person’s life. A study showed that being proud of oneself and perceiving one’s efforts as effective were the employees’ most important motivational factors [15]. Studies have shown that work motivation influences work performance and the outcomes of work in health care [1] For instance, a study, which focused on nurses, indicated that highly motivated nurses produced a more satisfactory outcome compared to the poorly motivated nurses [42]. Therefore, a person may be more willing to get involved in work if he believes that his efforts improve his/her performance [43].

A comparison between the nurses who worked in emergency departments and the nurses who worked in the other departments revealed that nurses had a high level of CPR motivation in terms of feelings of achievement, chances of success, perceived importance, and beliefs. Moreover, the results of the regression model showed that emergency department nurses had higher scores in the dimensions of feeling of achievement, high chances of success, low chances of success, perceived importance, and beliefs. The results of a study indicated that the respondents who worked in high-risk areas for cardiac arrest such as emergency department and/or were nursing personnel working continuously in close contact with patients were more motivated to learn CPR compared to the other hospital personnel [44]. Furthermore, the results of another study in Iran showed that the motivation of more than 65% of the nurses in emergency department ranged from intermediate level to high level [45]. The results of the study that was conducted by Suryandari et al. indicated that the motivation level of the majority of emergency nurses was high [43].

Several factors influence the different rates of cardiac arrest cases in emergency department patients. One of these factors in emergency departments is the cardiopulmonary arrest patients’ acute heart problems which lead to reversible traumas. Most of the cardiac arrests in the other departments stem from patients’ underlying diseases. Nonetheless, nurses’ experiences of the failure of cardiac resuscitation have reduced their motivation for resuscitating patients since they consider resuscitation to be a futile endeavor. Furthermore, the results of the studies have indicated that a patient’s underlying diseases are one of the motivating factors that prompt the nurses to perform cardiopulmonary resuscitation [46]. Consequently, the emergency department nurses’ attitudes toward the patients’ reversibility and their lack of knowledge about the patients’ underlying diseases have increased their motivation for treating arrest patients in emergency departments in comparison to the other departments. We can attribute the difference between the results of this study and the results of the study that was conducted by Assaroudi et al. [18] to their different research environments. The research environment of this study was one of the largest trauma centers in Iran.

Moreover, this study showed that emergency nurses’ motivation in the dimensions of perceived importance and beliefs was higher than the nurses’ motivation in the other departments. Nurses’ motivation is higher in the emergency department in comparison to the other departments. Consequently, it is essential to persuade the nurses in the other departments to attempt to restore the patients to life. Despite the obstacles and problems, nurses make an effort to perform cardiopulmonary resuscitation in a satisfactory way. It can be stated that nurses in the emergency department place a high value on saving patients’ lives. Additionally, some studies have shown that valuable work increases individuals’ motivation [31]. Considering the dimension of beliefs, emergency department nurses’ high motivation scores indicated that they hoped to restore the patient to life. Moreover, they were motivated to perform CPR based on the belief that God determines the time of death and that the resuscitator is under divine guidance. In this regard, the results of the studies showed that nurses’ beliefs affected their work [41]. Furthermore, higher work motivation makes a person more enthusiastic to provide the best service in order to improve his/her performance [43].

In addition, the study demonstrated that nurses who had a bachelor’s degree and had the experience of attending CPR workshops were more motivated in the dimension of understanding the importance of CPR compared to the nurses who had a master’s degree. Based on the results, health workers who had received some form of training in the preceding 12 months were more likely to have a higher motivation score [47]. Literature has shown that in-service training may be a motivating factor in health workers’ performance and may be as effective as a focus on the nurses’ higher wages [48].

It appears that the nurses with a bachelor’s degree exert greater effort to save the patients’ lives t during CPR. This difference may stem from the nurses’ higher job satisfaction compared to the postgraduates, which led to the nurses’ higher motivation. According to Baghshykhi et al. [41], nurses’ dissatisfaction may negatively affect their job satisfaction. Moreover, the high motivation score of the nurses who had the experience of participating in CPR workshops can be explained by the fact that participation in CPR workshops increased the nurses’ belief in the effectiveness of CPR and their respect for it. The results of the study which was ducted by Vali et al.‘s indicated that participation in training workshops increased the importance of CPR [49].

According to the results of the present study, there was a weak inverse relationship between the nurses’ participation in CPR frequency and their motivation in terms of their feelings of achievement, low chance of success, perceived importance, and beliefs. Furthermore, the results of the regression model showed that the frequency of participation in CPR had a negative effect on the score of the dimension of beliefs. That is, nurses’ participation in more resuscitation cases decreased their motivation. This result is in contrast with the results of the study that was conducted by Assaroudi et al. [18], and indicated that nurses’ motivation was not affected by the number of times they participated in cardiopulmonary resuscitation. Moreover, another study showed that years of experience was associated with motivation [1, 42]. However, a study that focused on nurses /midwives in Turkey found that the increase in the nurses’ experience was accompanied by a decrease in their job satisfaction and motivation. This result can be related to the increase in nurses’ family life responsibilities and their inability to derive satisfaction and from work [50]. This difference may be related to the difference in the research areas of these studies. A number of studies have shown that specialized training can increase internal and external work motivation [31]. In contrast to the results of the present study, Assaroudi et al. found that nurses’ motivation was not affected by CPR training [18]. Considering the results of Assaroudi et al. s’ study [18], it can be concluded that the specialized CPR rounds in their study environment did not necessarily improve their perception of their competence and capability and did not affect their motivation. Likewise, several studies have concluded that the continuous education programs in hospitals and the adult learning principles are disregarded [51]. In summary, nurses’ motivation to start or continue CPR decreases over time as a result of the unsuccessful results of most of CPRs. According to the results of Adib-Hajibagheri et al.‘s study, only 19.9% of patients who received CPR were restored to life. Moreover, only 5.6% of them were discharged [52]. In this situation, nurses feel that their efforts are not successful. Therefore, they lose motivation to perform CPR in the future. Additionally, Assaroudi et al. noted that a high likelihood of resuscitation success increased nurses’ motivation to resuscitate the patients. That is, the decrease in nurses’ motivation stemmed from the small chance of patients’ resuscitation success [18].

The results of the multiple linear regression model showed that the male participants’ scores of facilitators of resuscitation, and high chances of success were lower than that of female nurses. Moreover, based on the results of the regression model, the years of membership in the CPR team had a positive effect on the participants’ scores of feeling of achievement and high chances of success. The results of a study indicated that sociodemographic variables were important predictors of the changeability of the internal motivation factor score [53]. The results of a quantitative study, which was conducted in Iran, showed that sex, academic degree, years of experience and marital status were important variables that affected motivation factors [54]. However, few studies have claimed that, while male nurses’ motivation is influenced by rewards, morale, recognition, supervision and management, and communication, the female nurses’ motivation is affected by non-financial rewards [1, 42]. Nevertheless, based on the results of another study, female employees considered remuneration to be a more motivating factor in comparison with the male employees [55]. Similarly, another study, which focused on health workers, reported that female respondents had the highest motivation scores [47]. It seems that one of the reasons for the increase in women’s motivation compared to men is their higher job satisfaction in comparison with men. Research has shown that men are more motivated by higher wages and prestigious jobs. On the other hand, women are more concerned with their job security and the community value of their job [41].

### Limitations of the study

One of the limitations of this study stemmed from the fact that the results were limited to only one university center. This issue reduced the generalizability of the findings. Another limitation of this study was the lack of similar studies in this field. Therefore, the study attempted to use similar results from unrelated studies as a basis for comparison and interpretation. The results of this study showed that there is a need for more similar studies in other medical centers. These studies can provide more generalizable results. Moreover, the future studies need to investigate the factors that influence nurses’ motivation for performing cardiopulmonary resuscitation in a more comprehensive way.

## Conclusion

Based on the results of this study, it can be concluded that nurses are mainly motivated to perform CPR when they feel successful. Moreover, their motivation is negatively affected by their feelings of low chances of their success. In light of these results, health managers should identify the factors that reduce the chances of resuscitation success and have to develop strategies to eliminate these factors. These measures can increase nurses’ motivation, which is negatively affected by the ineffectiveness and the likelihood of unsuccessful resuscitation. Furthermore, nurses in the medical department had the lowest motivation for performing CPR. Therefore, it is necessary to investigate the factors that influence the outcome of CPR. Taking part in the CPR workshops increased nurses’ motivation in only two dimensions: perceived importance and having positive beliefs in CPR. Moreover, the study showed that there was a negative relationship between the number of times nurses performed CPR and their motivation. This finding implies that nurses became less motivated as they performed CPR on more individuals. Consequently, health managers must investigate the relevant factors in a more comprehensive way.

## Relevance for clinical practice

It is therefore necessary to plan and to conduct CPR workshops to ameliorate the nurses’ sense of success, high chance of success, sense of responsibility, and job satisfaction. This study seems to support the need for continuous and systematic refresher training for facilitating the nurses’ skill development and increasing their motivation.

## Data Availability

The datasets used and/or analyzed during the current study are available from the corresponding author upon reasonable request.

## Abbreviations

*CPR*: Cardiopulmonary Resuscitation
*CPRMS*: Cardiopulmonary Resuscitation Motivation Scale
*CI*: Confidence Interval
Std: Standard Deviation
B: Beta

## References

[1] Baljoon RA, Banjar HE, Banakhar MA (2018). Nurses’ work motivation and the factors affecting it: a scoping review. Int J Nurs Clin Practices.

[2] Dagher G, El-Farr H. Herzberg Motivation Hygiene Theory. 2023.

[3] Dianati M, Azizi-Fini I, Oghalaee Z, Gilasi H, Savari F (2019). The impacts of nursing staff education on perceived abuse among hospitalized elderly people: a field trial. Nurs Midwifery Stud.

[4] Rodriguez FS, Martin KG. Utilizing motivation-Hygiene Theory to improve satisfaction of registered nurses in the Intensive Care Unit of a. Critical Care Hospital; 2022.

[5] Dashti Kalantar R, Asadizaker M, Azizi-Fini I, Yadollahi S (2023). Perceived stress and Anxiety of Healthcare Providers before and after a Hospital Accreditation Program in Ahvaz City, Iran. J Client-Centered Nurs Care.

[6] Gawel JE (2019). Herzberg’s theory of motivation and Maslow’s hierarchy of needs. Practical Assess Res Evaluation.

[7] Alshmemri M, Shahwan-Akl L, Maude P (2017). Herzberg’s two-factor theory. Life Sci J.

[8] Yu L-C, Hsu C-L (2022). Understanding users’ urge to Post Online reviews: a study based on existence, relatedness, and Growth Theory. SAGE Open.

[9] Ryan JC (2023). Equity theory in action: how to attract locals into nursing jobs. Manag Decis.

[10] Hewett R, Shantz A, Mundy J, Alfes K (2018). Attribution theories in human resource management research: a review and research agenda. Int J Hum Resource Manage.

[11] De Simone S (2015). Expectancy value theory: motivating healthcare workers. Am Int J Contemp Res.

[12] Opoku FK. The practical reality of goal setting theory: evidence from the performance of nurses in the sunyani regional hospital of Ghana. 2016.

[13] Addiarto W, Yunita R (2023). Strategies to improve the quality of cardiopulmonary resuscitation (CPR): a literature review. Nurs UPDATE: Jurnal Ilmiah Ilmu Keperawatan.

[14] Farquharson B, Dixon D, Williams B, Torrens C, Philpott M, Laidlaw H, McDermott S (2023). The psychological and behavioural factors associated with laypeople initiating CPR for out-of-hospital cardiac arrest: a systematic review. BMC Cardiovasc Disord.

[15] Afolabi A, Fernando S, Bottiglieri T (2018). The effect of organisational factors in motivating healthcare employees: a systematic review. Br J Healthc Manage.

[16] Silverplats J, Strömsöe A, Äng B, Södersved Källestedt M-L (2022). Attitudes towards cardiopulmonary resuscitation situations and associations with potential influencing factors—A survey among in-hospital healthcare professionals. PLoS ONE.

[17] Nolan RP, Wilson E, Shuster M, Rowe BH, Stewart D, Zambon S. Readiness to perform cardiopulmonary resuscitation: an emerging strategy against Sudden Cardiac Death. Psychosom Med. 1999;61(4). 10.1097/00006842-199907000-00020 10443764

[18] Assarroudi A, Nabavi FH, Ebadi A (2019). Motivation for cardiopulmonary resuscitation: scale development and psychometric analysis. Int Emerg Nurs.

[19] Bhanji F, Donoghue AJ, Wolff MS, Flores GE, Halamek LP, Berman JM (2015). Part 14: education: 2015 American Heart Association guidelines update for cardiopulmonary resuscitation and emergency cardiovascular care. Circulation.

[20] Nazri Panjaki A, Salari N, Khoshfetrat M. The relationship between working shifts and the success rate of cardio-pulmonary resuscitation in emergencies and wards. medical journal of mashhad university of medical sciences. 2017;60(4):610– 7.10.22038/mjms.2017.10187.

[21] Andersen LW, Holmberg MJ, Berg KM, Donnino MW, Granfeldt A (2019). In-hospital cardiac arrest: a review. JAMA.

[22] Finke S-R, Schroeder DC, Ecker H, Wingen S, Hinkelbein J, Wetsch WA (2018). Gender aspects in cardiopulmonary resuscitation by schoolchildren: a systematic review. Resuscitation.

[23] Jones BA, Thornton MA, Heid CA, Burke KL, Scrushy MG, Abdelfattah KR (2023). Survival after multiple episodes of cardiac arrest. Heart Lung.

[24] Zhu A, Zhang J (2016). Meta-analysis of outcomes of the 2005 and 2010 cardiopulmonary resuscitation guidelines for adults with in-hospital cardiac arrest. Am J Emerg Med.

[25] Schluep M, Gravesteijn BY, Stolker RJ, Endeman H, Hoeks SE (2018). One-year survival after in-hospital cardiac arrest: a systematic review and meta-analysis. Resuscitation.

[26] Assarroudi A, Heshmati Nabavi F, Ebadi A, Esmaily H (2017). Professional rescuers’ experiences of motivation for cardiopulmonary resuscitation: a qualitative study. Nurs Health Sci.

[27] Assarroudi A, Nabavi FH, Ebadi A, Esmaily H (2017). Do-not-resuscitate order: the experiences of Iranian cardiopulmonary resuscitation team members. Indian J Palliat Care.

[28] Riggs M, Franklin R, Saylany L (2019). Associations between cardiopulmonary resuscitation (CPR) knowledge, self-efficacy, training history and willingness to perform CPR and CPR psychomotor skills: a systematic review. Resuscitation.

[29] Khan SB, Proverbs DG, Xiao H (2022). The motivation of operatives in small construction firms towards health and safety–A conceptual framework. Eng Constr Architectural Manage.

[30] Fallahi M, Mahdavikian S, Abdi A, Borhani F, Taghizadeh P, Hematpoor B (2018). Nurses and physicians’ viewpoints about decision making of do not attempt resuscitation (DNAR). Multidisciplinary Respiratory Med.

[31] Toode K, Routasalo P, Helminen M, Suominen T (2015). Hospital nurses’ work motivation. Scand J Caring Sci.

[32] Umuhoza C, Chen L, Unyuzumutima J, McCall N (2021). Impact of structured basic life-support course on nurses’ cardiopulmonary resuscitation knowledge and skills: experience of a paediatric department in low-resource country. Afr J Emerg Med.

[33] Kleinman ME, Brennan EE, Goldberger ZD, Swor RA, Terry M, Bobrow BJ (2015). Part 5: adult basic life support and cardiopulmonary resuscitation quality: 2015 American Heart Association guidelines update for cardiopulmonary resuscitation and emergency cardiovascular care. Circulation.

[34] Fallahi M, Banaderakhshan H, Abdi A, Borhani F, Kaviannezhad R, Karimpour HA. The Iranian physicians attitude toward the do not resuscitate order. J Multidisciplinary Healthc. 2016:279–84. 10.2147/JMDH.S105002 PMC4935161 27418832

[35] Pei L, Liang F, Sun S, Wang H, Dou H (2019). Nursing students’ knowledge, willingness, and attitudes toward the first aid behavior as bystanders in traffic accident trauma: a cross-sectional survey. Int J Nurs Sci.

[36] Jiang Y, Wu B, Long L, Li J, Jin X (2020). Attitudes and willingness toward out-of-hospital cardiopulmonary resuscitation: a questionnaire study among the public trained online in China. BMJ open.

[37] Pivač S, Gradišek P, Skela-Savič B (2020). The impact of cardiopulmonary resuscitation (CPR) training on schoolchildren and their CPR knowledge, attitudes toward CPR, and willingness to help others and to perform CPR: mixed methods research design. BMC Public Health.

[38] Huang EP-C, Chiang W-C, Hsieh M-J, Wang H-C, Yang C-W, Lu T-C (2019). Public knowledge, attitudes and willingness regarding bystander cardiopulmonary resuscitation: a nationwide survey in Taiwan. J Formos Med Assoc.

[39] Sheikholeslami R (2016). Investigation of the content and process based factors affecting motivation of Public librarians of Iran (Case Study of Fars Province). Res Inform Sci Public Libr.

[40] Yadollahi S, Ebadi A, Asadizaker M (2020). Measuring Cultural competence in nursing: a review study. J Client-Centered Nurs Care.

[41] Baghshykhi F, Rahimi H, Azizi-Fini I, Haji-Jafari S, Izadi-Dastgerdi E (2020). Job characteristics and work adjustment among Iranian nurses: a correlational study. Nurs Midwifery Stud.

[42] Abu Yahya O, Ismaile S, Allari RS, Hammoudi BM, editors. Correlates of nurses’ motivation and their demographic characteristics. Nursing forum. Wiley Online Library; 2019. 10.1111/nuf.12291 30508252

[43] Suryandari D, Susanto ER. The effect of nurse competence, motivation, and workload on the performance of emergency room (ER) nurse in Regional hospitals of Yogyakarta. Eur J Bus Manage. 2018;10.

[44] Sánchez García AB, Fernández Alemán JL, Alonso Pérez N, Hernandez Hernández I, Navarro Valverde R, Rosillo Castro D (2015). Assessment of the knowledge level and its relevance in terms of CPR in medical personnel of the hospital emergency medical system of the Autonomous Community of the region of Murcia. Enferm Glob.

[45] Sheikhbardsiri H, Khademipour G, Nekoei-Moghadam M, Aminizadeh M (2018). Motivation of the nurses in pre-hospital emergency and educational hospitals emergency in the southeast of Iran. Int J Health Plann Manag.

[46] Toghraie M, Pourafzali SM. Study of the Barriers to the Success of Cardiopulmonary Resuscitation Team in Selected Hospitals of Isfahan City, Iran, in Years 2017–2018 from the Perspective of Physicians and Nurses. Journal of Isfahan Medical School. 2019;37(528):548– 55.10.22122/jims.v37i528.11624.

[47] Mutale W, Ayles H, Bond V, Mwanamwenge MT, Balabanova D (2013). Measuring health workers’ motivation in rural health facilities: baseline results from three study districts in Zambia. Hum Resour Health.

[48] Okello DR, Gilson L (2015). Exploring the influence of trust relationships on motivation in the health sector: a systematic review. Hum Resour Health.

[49] Leila V, Amir-Hosin F, Mehdi R-A, Seyed-Hosein S-A (2015). Motivational factors affecting the participation of nurses in in-service training programs: a case study of nurses working in public-educational hospitals, Kerma University of Medical Sciences. J Healthc Manag.

[50] Ertekin Pınar Ş, Ucuk S, Duran Aksoy Ö, Yurtsal Z, Cesur B, İçer Yel H. Job satisfaction and Motivation Levels of Midwives/Nurses Working inFamily Health Centres: a Survey from Turkey. Int J Caring Sci. 2017;10(2).

[51] Parviniannasab AM, Taheri NK (2022). Effectiveness of Nursing Continues Education Program in Iran: a narrative review. J Nurs Educ.

[52] Adib-HajBagheri Mohsen A, Hossein, Seyyed-Gholamabas M (2010). Success of cardiopulmonary resuscitation operations in Kashan hospitals. Res Med Sci.

[53] Weldegebriel Z, Ejigu Y, Weldegebreal F, Woldie M (2016). Motivation of health workers and associated factors in public hospitals of West Amhara, Northwest Ethiopia. Patient Prefer Adherence.

[54] Heidarian AR, Jafari Kelarijani SE, Jamshidi R, Khorshidi M (2015). The relationship between demographic characteristics and motivational factors in the employees of social security hospitals in Mazandaran. Casp J Intern Med.

[55] Zarei E, Najafi M, Rajaee R, Shamseddini A. Determinants of job motivation among frontline employees at hospitals in Tehran. Electron Physician. 2016;8(4):2249– 54.10.19082/2249. 10.19082/2249 PMC4886566 27280000

